# Association Between Variants in Calcineurin Inhibitor Pharmacokinetic and Pharmacodynamic Genes and Renal Dysfunction in Adult Heart Transplant Recipients

**DOI:** 10.3389/fgene.2021.658983

**Published:** 2021-04-01

**Authors:** Kris Oreschak, Laura M. Saba, Nicholas Rafaels, Amrut V. Ambardekar, Kimberly M. Deininger, Robert L. Page, JoAnn Lindenfeld, Christina L. Aquilante

**Affiliations:** ^1^Department of Pharmaceutical Sciences, University of Colorado Skaggs School of Pharmacy and Pharmaceutical Sciences, Aurora, CO, United States; ^2^Division of Biomedical Informatics and Personalized Medicine, Department of Medicine, University of Colorado School of Medicine, Aurora, CO, United States; ^3^Division of Cardiology, University of Colorado School of Medicine, Aurora, CO, United States; ^4^Division of Clinical Pharmacy, University of Colorado Skaggs School of Pharmacy and Pharmaceutical Sciences, Aurora, CO, United States; ^5^Division of Cardiology, Vanderbilt University Medical Center, Nashville, TN, United States

**Keywords:** renal dysfunction, estimated glomerular filtration rate, heart transplant, pharmacogenetics, pharmacogenomics, calcineurin inhibitor, single nucleotide variant

## Abstract

**Background**: The goal of the study was to assess the relationship between single nucleotide variants (SNVs) in calcineurin inhibitor (CNI) pharmacokinetic and pharmacodynamic genes and renal dysfunction in adult heart transplant (HTx) recipients.

**Methods**: This retrospective analysis included *N* = 192 patients receiving a CNI at 1-year post-HTx. Using a candidate gene approach, 93 SNVs in eight pharmacokinetic and 35 pharmacodynamic genes were chosen for investigation. The primary outcome was renal dysfunction 1-year after HTx, defined as an estimated glomerular filtration rate (eGFR) <45 ml/min/1.73m^2^.

**Results:** Renal dysfunction was present in 28.6% of patients 1-year after HTx. Two SNVs [transforming growth factor beta 1 (*TGFB1*) rs4803455 C > A and phospholipase C beta 1 (*PLCB1*) rs170549 G > A] were significantly associated with renal dysfunction after accounting for a false discovery rate (FDR) of 20%. In a multiple-SNV adjusted model, variant A allele carriers of *TGFB1* rs4803455 had lower odds of renal dysfunction compared to C/C homozygotes [odds ratio (OR) 0.28, 95% CI 0.12–0.62; *p* = 0.002], whereas *PLCB1* rs170549 variant A allele carriers had higher odds of the primary outcome vs. patients with the G/G genotype (OR 2.66, 95% CI 1.21–5.84, *p* = 0.015).

**Conclusion**: Our data suggest that genetic variation in *TGFB1* and *PLCB1* may contribute to the occurrence of renal dysfunction in HTx recipients receiving CNIs. Pharmacogenetic markers, such as *TGFB1* rs4803455 and *PLCB1* rs170549, could help identify patients at increased risk of CNI-associated renal dysfunction following HTx, potentially allowing clinicians to provide more precise and personalized care to this population.

## Introduction

Renal dysfunction is a major challenge following heart transplantation, with over 50% of patients experiencing some degree of renal impairment at 1-year post-transplant (Hamour et al., 2009; Navarro-Manchon et al., 2010). Renal dysfunction is associated with significant morbidity and a 4-fold increase in the risk of mortality in heart transplant recipients (van Gelder et al., 1998; Ojo et al., 2003; Alba et al., 2016; Kolsrud et al., 2018). The development of renal dysfunction is multifactorial, with contributing factors such as older age at transplant, other comorbidities (e.g., hypertension and diabetes mellitus), and long-term use of nephrotoxic drugs such as calcineurin inhibitors (CNIs; Wilkinson and Cohen, 1999; Ojo, 2007).

Calcineurin inhibitors remain the cornerstone of maintenance immunosuppressant therapy following heart transplantation, with 98% of patients receiving a CNI, most commonly tacrolimus, at 1-year post-transplant (Khush et al., 2019). Although excellent at preventing rejection, CNIs are associated with the development of chronic renal dysfunction, the exact mechanism of which is unknown. Importantly, systemic CNI exposure is not predictive of renal impairment, as renal dysfunction often occurs despite CNI trough blood levels being within goal range (van Gelder et al., 1998; Wilkinson and Cohen, 1999).

The field of pharmacogenetics seeks to characterize the impact of genetic variation, including single nucleotide variants (SNVs), on drug disposition, response, and adverse effects (Roden et al., 2019). To date, only two pharmacogenetic studies have comprehensively investigated the association between SNVs and renal function in heart transplant recipients; however, CNI prescribing patterns in those studies do not reflect contemporary clinical practice, with only 21–36% of patients on tacrolimus therapy (Lachance et al., 2012; Asleh et al., 2018). Given the deleterious consequences of renal dysfunction after heart transplant and the contribution of CNIs to its development, there is a critical need to identify novel, patient-specific factors that increase the risk of this adverse effect. As such, the goal of this study was to evaluate the association between SNVs in key CNI pharmacokinetic and pharmacodynamic genes and renal dysfunction in heart transplant recipients taking CNIs at 1-year post-transplant.

## Materials and Methods

### Study Population

The data for this retrospective cohort study were obtained from heart transplant patients who had participated in a parent pharmacogenomic study at the University of Colorado. The overarching goal of the parent study was to assess the association between SNVs and renal dysfunction in patients receiving CNIs in the first 5 years post-heart transplant. Patients were included in the parent study if they had received a heart-only transplant, were at least 18 years old at the time of transplant, and were prescribed a CNI post-transplant. Patients were excluded if they underwent multi-organ transplant or did not provide informed consent. In total, 253 adult heart transplant recipients were enrolled in the parent study at routine clinic visits, where a mouthwash or blood sample was collected for genetic analysis. Demographic and clinical data were retrospectively collected from participants’ medical records at the following time points: pre-transplant, transplant discharge, 6 months post-transplant, and annually from 1 to 5-years post-transplant. The parent study was approved by the Colorado Multiple Institutional Review Board and all participants provided written, informed consent.

Here, we describe analyses for the 1-year post-transplant time point. Parent study participants were included in these analyses if they received a CNI at the 1-year post-transplant clinic visit and had at least one estimated glomerular filtration rate (eGFR) measurement available in their medical records in the 3 months prior to transplant and at 1-year after transplantation (±2 months). Participants were excluded if they did not receive a CNI at the 1-year post-transplant clinic visit or if eGFR measurements were unavailable. Those transplanted at hospitals other than the University of Colorado were included if relevant clinical data were available in their medical records. The number of participants from the parent study who met eligibility criteria for the 1-year analysis was *N* = 192.

### Study Outcomes

The primary outcome of the study was renal dysfunction (yes/no), defined as an eGFR <45 ml/min/1.73 m^2^ [i.e., grade 3B or worse per the Kidney Disease Outcomes Quality Initiative (KDOQI) guidelines] calculated using the Modification of Diet in Renal Disease (MDRD) formula, at 1-year post-transplant (National Kidney Foundation, 2002; Zalawadiya et al., 2017). The single eGFR measurement closest to the 1-year transplant anniversary (±2 months) was used to assess renal dysfunction. The secondary outcomes were the continuous measurements of eGFR 1-year after transplantation and change in eGFR from pre-transplant to 1-year post-transplant. CNI exposure was defined as being prescribed either cyclosporine or tacrolimus at the 1-year post-transplant clinic visit.

### Genotyping, Quality Control, and Imputation

Genomic DNA was isolated from either buccal cells in mouthwash samples or leukocytes in whole blood using a commercially available kit (QIAamp® DNA Mini Kit; Qiagen, Valencia, CA, United States). Genotyping was performed on a customized version of the Illumina® Expanded Multi-Ethnic Global Array (CU-MEGA^EX^; Infinium, 2015). The CU-MEGA^EX^ contains over 2 million genetic variants, including over 20,000 drug metabolism and excretion markers, and incorporates content from Phase 3 of the 1,000 Genomes Project, the Consortium on Asthma among African-ancestry Populations in the Americas (CAAPA), and Population Architecture using Genomics and Epidemiology (PAGE; Matise et al., 2011; 1000 Genomes Project Consortium, 2015; Mathias et al., 2016).

Genotype quality control was performed using PLINK v1.90 (Chang et al., 2015). Patients with array-wide call rates <90% were excluded from the analysis. Next, we selected a set of high-quality SNVs to determine heterozygosity, relatedness, and principal components (PCs). High-quality SNVs were defined as having Hardy-Weinberg equilibrium values of *p* > 1 × 10^−6^, call rates >95%, and minor allele frequencies >1%. Patients with excessively high heterozygosity rates (i.e., >3 SD above the mean) and one sibling from a pair of related samples (defined as a pairwise identity by descent >0.185) were excluded from the analysis. Using the scree test, the top four PCs were selected for inclusion in adjusted models based on the “elbow” of the scree plot (Supplementary Figure S1). Variants not present on the CU-MEGA^EX^ were imputed using the Michigan Imputation server (Das et al., 2016). Imputation was performed using Minimac4, with the Haplotype Reference Consortium version r1.1 2016 serving as the reference panel (McCarthy et al., 2016). SNVs with imputation values of *r*^2^ > 0.6, minor allele frequencies >5%, and Hardy-Weinberg equilibrium values of *p* > 1 × 10^−6^ in Americans of European ancestry were considered for analysis.

Following quality control and imputation procedures, we employed a candidate gene approach, whereby we selected SNVs in CNI pharmacokinetic and pharmacodynamic genes. Candidate genes were chosen based on the following criteria: (1) genes involved in CNI clinical pharmacology and renal pathophysiology; (2) genes previously associated with renal function in transplant populations; and (3) significant predictors of non-transplant chronic kidney disease in genome wide association studies (Baan et al., 2000; Lacha et al., 2001; van de Wetering et al., 2006; Kottgen et al., 2008; Smith et al., 2008; Jacobson et al., 2012; Lachance et al., 2012). Within these genes, we chose SNVs based on functional or clinical significance, annotation in pharmacogenomic databases,^1^ and/or HapMap tagging variants. In total, 93 SNVs were evaluated – 22 SNVs in eight CNI pharmacokinetic genes and 71 SNVs in 35 CNI pharmacodynamic genes (Supplementary Table S1).

### Statistical Analyses

Descriptive data are presented as mean ± SD or *n* (%). The Shapiro-Wilk test was used to assess for normality; non-normally distributed data were log-transformed prior to analysis and back-transformed for presentation. Relationships between demographic and clinical variables [i.e., age at transplant, sex, pre-transplant eGFR, pre-transplant renal dysfunction (eGFR <45 ml/min/1.73 m^2^; yes/no), heart failure etiology [ischemic cardiomyopathy (CM) vs. other], pre-transplant left ventricular assist device (LVAD; yes/no), pre-transplant hypertension (yes/no), pre-transplant diabetes (yes/no), body mass index (BMI) 1-year post-transplant, transplant era, type of CNI used 1-year post-transplant (i.e., cyclosporine vs. tacrolimus), and angiotensin-converting enzyme (ACE) inhibitor or angiotensin II receptor blocker (ARB) prescribed 1-year post-transplant (yes/no)] and the primary and secondary outcomes were assessed using Pearson’s correlation coefficients, with significant covariates (*p* < 0.05) included in the relevant adjusted analyses. Patients were divided into one of three transplant eras, as defined by the most recent International Society for Heart and Lung Transplantation annual report, based on their date of transplant (era 1, 1989–2001; era 2, 2002–2009; era 3, 2010–2016; Khush et al., 2019). To account for potential population stratification, the top four PCs were included in all adjusted analyses performed in the entire cohort. SNVs were analyzed as wild-type (WT) homozygotes vs. variant carriers using a dominant model.

For the primary outcome, univariate analyses were performed using Fisher’s exact tests to identify SNVs associated with renal dysfunction (yes/no). SNVs that were significant after accounting for a false discovery rate (FDR) of 20% were included in a multiple-SNV logistic regression model, adjusting for pertinent covariates and the top four PCs. As exploratory analyses, SNVs suggestively associated with the primary outcome in the univariate analyses [unadjusted *p* < 0.05, but not significant after accounting for multiple comparisons (FDR of 20%)], were investigated in single-SNV logistic regression models, adjusting for relevant covariates and the top four PCs. Univariate analyses to assess the relationship between SNVs and each secondary outcome were performed using *t*-tests. Single-SNV analyses were carried out using linear models and included SNVs suggestively associated [i.e., unadjusted *p* < 0.05 but not significant following adjustment for multiple comparisons (FDR of 20%)] with the secondary outcomes plus relevant covariates and the top four PCs.

For all primary and secondary outcomes, SNV analyses including relevant covariates were also performed in non-Hispanic Americans of European ancestry to assess whether significant and suggestive SNVs remained associated with each outcome in this subgroup. Statistical analyses were performed using R version 3.5.2 (R Foundation for Statistical Computing, Vienna Austria).

## Results

### Patient Characteristics

Of the 253 participants enrolled in the parent study, 192 participants met the inclusion and exclusion criteria for this 1-year post-transplant analysis. The most common reason participants from the parent study were excluded was the absence of an eGFR measurement prior to transplant (*n* = 44). Characteristics of the 192 participants are shown in Table 1 and described as follows. Most participants were transplanted between May 1989 and August 2016 at the University of Colorado (96.4%). The cohort was primarily of European ancestry (79.2%) and male (77.1%), with a mean ± SD age at transplant of 49 ± 12 years. Tacrolimus (53.1%) was the most frequent CNI used 1-year post-transplant. Pre-transplant hypertension and pre-transplant renal dysfunction were present in 33.3 and 20.3% of patients, respectively. Less than one-third of participants (27.6%) had an LVAD prior to transplant. Approximately two-thirds of patients (63.5%) were prescribed an ACE inhibitor or an ARB 1-year post-transplant.

**Table 1:** Participant demographic and clinical characteristics.

Characteristic	Entire cohort (*N* = 192)	No renal dysfunction at 1-year post-transplant (*N* = 137)	Renal dysfunction^a^ at 1-year post-transplant (*N* = 55)	*p*
Sex (male)	148 (77.1%)	110 (80.3%)	38 (69.1%)	0.128
Age at transplant (years)	49 ± 12	47 ± 13	55 ± 9	<0.001
**Race**				0.222
European American	152 (79.2%)	106 (77.4%)	46 (83.6%)	
African American	17 (8.9%)	15 (10.9%)	2 (3.6%)	
Asian	7 (3.6%)	6 (4.4%)	1 (1.8%)	
Other^b^	16 (8.3%)	10 (7.3%)	6 (10.9%)	
**Ethnicity**				1.000
Non-hispanic	172 (89.6%)	123 (89.8%)	49 (89.1%)	
Hispanic	20 (10.4%)	14 (10.2%)	6 (10.9%)	
**Reason for transplant**				0.957
Nonischemic CM	91 (47.4%)	67 (48.9%)	24 (43.6%)	
Ischemic CM	64 (33.3%)	44 (32.1%)	20 (36.4%)	
Valvular CM	9 (4.7%)	6 (4.4%)	3 (5.5%)	
Mixed etiology	8 (4.2%)	6 (4.4%)	2 (3.6%)	
Congenital CM	8 (4.2%)	5 (3.6%)	3 (5.5%)	
Other	12 (6.3%)	9 (6.6%)	3 (5.5%)	
Pre-transplant hypertension	64 (33.3%)	47 (34.3%)	17 (30.9%)	0.736
Pre-transplant diabetes	25 (13.0%)	16 (11.7%)	9 (16.4%)	0.477
Pre-transplant eGFR (ml/min/1.73 m^2^)	65 ± 27	71 ± 28	51 ± 16	<0.001
Pre-transplant renal dysfunction^a^	39 (20.3%)	21 (15.3%)	18 (32.7%)	0.010
Pre-transplant LVAD	53 (27.6%)	39 (28.5%)	14 (25.5%)	0.724
Body mass index (kg/m^2^)^c^	27.0 ± 4.8	26.5 ± 4.6	28.1 ± 5.3	0.056
eGFR 1-year post-transplant (ml/min/1.73 m^2^)	55 ± 20	63 ± 19	37 ± 6	<0.001
Change in eGFR (ml/min/1.73 m^2^)^d^	−10 ± 21	−8 ± 23	−14 ± 15	0.037
**Calcineurin inhibitor^c^**				0.004
Tacrolimus	102 (53.1%)	82 (59.9%)	20 (36.4%)	
Cyclosporine	90 (46.9%)	55 (40.1%)	35 (63.6%)	
Tacrolimus total daily dose (mg/day)^c^	6.3 ± 4.8	6.5 ± 5.1	5.3 ± 3.2	0.291
Tacrolimus trough level (ng/ml)^c^	11.4 ± 3.8	11.7 ± 3.8	9.8 ± 3.6	0.019
Cyclosporine total daily dose (mg/day)^c^	278 ± 96	284 ± 92	269 ± 102	0.339
Cyclosporine trough level (ng/ml)^c^	238 ± 101	242 ± 109	232 ± 89	0.630
**Antiproliferative agent^c^**				0.011
Mycophenolate	153 (79.7%)	115 (83.9%)	38 (69.1%)	
Azathioprine	28 (14.6%)	14 (10.2%)	14 (25.5%)	
None	11 (5.7%)	8 (5.8%)	3 (5.5%)	
ACE inhibitor/ARB^c^	122 (63.5%)	88 (64.2%)	34 (61.8%)	0.744
**Transplant era**				0.031
Era 1: 1989–2001	60 (31.3%)	35 (25.5%)	25 (45.5%)	
Era 2: 2002–2009	58 (30.2%)	45 (32.8%)	13 (23.6%)	
Era 3: 2010–2016	74 (38.5%)	57 (41.6%)	17 (30.9%)	

Data are presented as mean ± SD or *N* (%). Percentages may not equal 100% due to rounding. ACE, angiotensin-converting enzyme; ARB, angiotensin II receptor blocker; CM, cardiomyopathy; eGFR, estimated glomerular filtration rate; and LVAD, left ventricular assist device.

a eGFR <45 ml/min/1.73 m^2^ calculated using the Modification of Diet in Renal Disease (MDRD) formula.

b Includes *n* = 15 Hispanics and *n* = 1 American Indian.

c At 1-year clinic visit.

d Change in eGFR from pre-transplant to 1-year post-transplant.

### Renal Dysfunction at 1-Year Post-transplant

Renal dysfunction was present in 28.6% of patients at 1-year post-transplant. Demographic and clinical variables associated with renal dysfunction 1-year post-transplant were the presence of pre-transplant renal dysfunction (*p* = 0.007), lower pre-transplant eGFR (*p* < 0.001), older age at transplant (*p* < 0.001), cyclosporine use at 1-year post-transplant (*p* = 0.003), and receiving a transplant in an earlier era (*p* = 0.021). Pre-transplant renal dysfunction (yes/no) was selected for inclusion in covariate-adjusted models rather than the continuous measurement of eGFR due to the dichotomous nature of the primary outcome (Oetjens et al., 2014). Transplant era remained significantly associated with renal dysfunction at the 1-year time point after adjusting for type of CNI prescribed (*p* = 0.035). Tacrolimus trough blood levels were significantly lower in patients with vs. without renal dysfunction at 1-year post-transplant (9.8 ± 3.6 ng/ml vs. 11.7 ± 3.8 ng/ml; *p* = 0.019), likely reflecting clinical intervention in response to decreased eGFR. Cyclosporine trough blood levels, tacrolimus total daily dose, and cyclosporine total daily dose did not differ significantly between the two groups at 1-year post-transplant.

Two SNVs [transforming growth factor beta 1 (*TGFB1*) rs4803455 C > A and phospholipase C beta 1 (*PLCB1*) rs170549 G > A] were significantly associated with renal dysfunction 1-year post-transplant in the univariate analyses after adjusting for an FDR of 20% (unadjusted *p* = 0.004 for both SNVs). Renal dysfunction was present in 43% of participants with the *TGFB1* rs4803455 C/C genotype vs. 22% of variant A carriers, and 20% of participants with the *PLCB1* rs170549 G/G genotype vs. 39% of variant A carriers. *TGFB1* rs4803455 and *PLCB1* rs170549 remained significantly associated with the primary outcome in a multiple-SNV covariate-adjusted logistic regression model (Table 2). Specifically, *TGFB1* rs4803455 variant A carriers had lower odds of renal dysfunction when compared to participants with the C/C genotype [odds ratio (OR) 0.28; 95% CI 0.12–0.62; *p* = 0.002]. In contrast, *PLCB1* rs170549 variant A allele carriers had higher odds of renal dysfunction than G/G homozygotes (OR 2.66; 95% CI 1.21–5.84; *p* = 0.015). Additionally, both SNVs were significantly associated with pre-transplant renal dysfunction, with this morbidity occurring in 30% of participants with the *TGFB1* rs4803455 C/C genotype vs. 16% of variant A carriers (*p* = 0.018), and 12% of participants with the *PLCB1* rs170549 G/G genotype vs. 30% of variant A carriers (*p* = 0.002). When limiting the multiple-SNV covariate-adjusted analysis to non-Hispanic Americans of European ancestry, both *TGFB1* rs4803455 C > A (OR 0.39; 95% CI 0.17–0.90; *p* = 0.027) and *PLCB1* rs170549 G > A (OR 2.52; 95% CI 1.10–5.77; *p* = 0.029) remained significantly associated with the outcome (Supplementary Table S2).

**Table 2:** Multiple-SNV adjusted model for SNVs significantly associated with renal dysfunction^a^ 1-year post-transplant.

Gene name	rs number	Chromosome	Alleles^b^	MAF (%)	Reference group	Adjusted^c^ odds ratio (95% CI)	Adjusted^c^ value of *p*
*TGFB1*	rs4803455	19	C > A	45	C/C	0.28 (0.12–0.62)	0.002
*PLCB1*	rs170549	20	G > A	28	G/G	2.66 (1.21–5.84)	0.015

MAF, minor allele frequency; PLCB1, phospholipase C beta 1; SNV, single nucleotide variant; and TGFB1, transforming growth factor beta 1.

a Defined as an eGFR <45 ml/min/1.73m^2^.

b Major > minor alleles, respectively.

c Model adjusting for pre-transplant renal dysfunction, age at transplant, cyclosporine use at 1-year post-transplant, transplant era, and the top four principal components (PCs).

Six SNVs in the univariate analyses were suggestively associated with renal dysfunction 1-year post-transplant but were not significant after accounting for an FDR of 20% (Table 3). As an exploratory analysis, each SNV was evaluated in a single-SNV logistic regression model, adjusting for pre-transplant renal dysfunction, age at transplant, type of CNI prescribed at 1-year post-transplant, transplant era, and the top four PCs. Five SNVs [calcium voltage-gated channel subunit alpha1 D (*CACNA1D*) rs893365 C > T, protein phosphatase 3 catalytic subunit gamma (*PPP3CC*) rs10108011 A > G, *PPP3CC* rs2461494 A > G, protein kinase AMP-activated non-catalytic subunit gamma 2 (*PRKAG2*) rs7805747 G > A, and nuclear receptor subfamily 3 group C member 2 (*NR3C2*) rs1490453 G > A] remained suggestively associated with the primary outcome in the single-SNV covariate-adjusted models. The odds of renal dysfunction were higher in variant carriers of *PPP3CC* rs10108011, *PPP3CC* rs2461494, and *PRKAG2* rs7805747, whereas the odds of renal dysfunction were lower in variant carriers of *CACNA1D* rs893365 and *NR3C2* rs1490453. When these five SNVs were evaluated in non-Hispanic Americans of European ancestry, only *CACNA1D* rs893365 and *PRKAG2* rs7805747 continued to be associated with lower and higher odds of renal dysfunction 1-year post-transplant, respectively, after adjusting for covariates (Supplementary Table S3).

**Table 3:** Unadjusted and adjusted single-SNV analyses for SNVs suggestively associated with renal dysfunction^a^ at 1-year post-transplant.

Gene name	rs number	Chromosome	Alleles^b^	MAF (%)	Reference group	Unadjusted odds ratio (95% CI)	Unadjusted value of *p*	Adjusted^c^ odds ratio (95% CI)	Adjusted^c^ value of *p*
*CACNA1D*	rs893365	3	C > T	49	CC	0.44 (0.22–0.89)	0.025	0.28 (0.12–0.69)	0.005
*PPP3CC*	rs10108011	8	A > G	42	AA	2.30 (1.13–4.68)	0.021	3.30 (1.39–7.82)	0.007
*PPP3CC*	rs2461494	8	A > G	20	AA	2.19 (1.16–4.15)	0.021	2.62 (1.24–5.54)	0.012
*PRKAG2*	rs7805747	7	G > A	24	GG	2.11 (1.12–3.98)	0.024	2.13 (1.03–4.39)	0.041
*NR3C2*	rs1490453	4	G > A	18	GG	0.47 (0.23–0.97)	0.043	0.44 (0.20–0.99)	0.047
*PLCB1*	rs227129	20	G > A	28	GG	1.98 (1.05–3.75)	0.039	1.89 (0.92–3.89)	0.083

CACNA1D, calcium voltage-gated channel subunit alpha1 D; MAF, minor allele frequency; NR3C2, nuclear receptor subfamily 3 group C member 2; PLCB1, phospholipase C beta 1; PPP3CC, protein phosphatase 3 catalytic subunit gamma; PRKAG2, protein kinase AMP-activated non-catalytic subunit gamma 2; and SNV, single nucleotide variant.

a Defined as an eGFR <45 ml/min/1.73m^2^.

b Major > minor alleles, respectively.

c Models adjusted for pre-transplant renal dysfunction, age at transplant, cyclosporine use at 1-year post-transplant, transplant era, and the top four PCs.

### eGFR 1-Year Post-transplant

**Figure 1:**
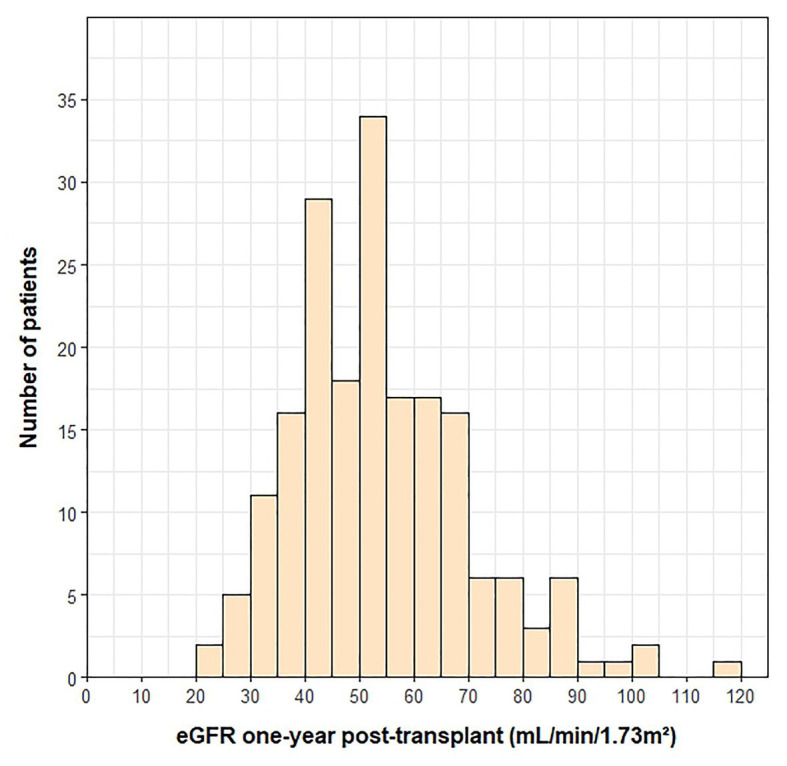
Histogram of eGFR measurements at 1-year post-transplant. One outlier (eGFR = 210) was not included on the histogram.

A histogram of the eGFR measurements closest to the 1-year transplant anniversary is shown in Figure 1. The mean ± SD time from the 1-year anniversary to the eGFR measurement was 10 ± 9 days. The mean eGFR 1-year post-transplant was 55 ± 20 ml/min/1.73 m^2^. Demographic and clinical variables associated with lower eGFR 1-year post-transplant were female sex (*p* = 0.018), presence of pre-transplant renal dysfunction (*p* < 0.001), lower pre-transplant eGFR (*p* < 0.001), older age at transplant (*p* < 0.001), cyclosporine use at 1-year post-transplant (*p* = 0.001), and higher BMI at 1-year post-transplant (*p* = 0.002). The measurement of pre-transplant eGFR was included as a covariate in adjusted analyses, as the outcome (i.e., eGFR 1-year post-transplant) was a continuous variable.

Five SNVs were suggestively associated with eGFR 1-year post-transplant (*p* < 0.05; Table 4), but they did not withstand correction for multiple testing (FDR of 20%). As an exploratory analysis, each SNV was evaluated in a single-SNV adjusted linear regression model, adjusting for the demographic and clinical variables above. Two SNVs in the protein phosphatase 3 regulatory subunit B alpha (*PPP3R1*) gene, rs875 A > G and rs12465425 T > G, remained suggestively associated with the outcome in single-SNV adjusted models, with variant G allele carriers having higher eGFR 1-year post-transplant compared to WT homozygotes for both SNVs. Neither of these two *PPP3R1* SNVs were suggestively associated with eGFR 1-year post-transplant in the single-SNV adjusted models when limiting the analysis to non-Hispanic Americans of European ancestry (Supplementary Table S4).

**Table 4:** Unadjusted and adjusted single-SNV analyses for SNVs suggestively associated with eGFR^a^ at 1-year post-transplant.

Gene name	rs number	Chromosome	Alleles^b^	MAF (%)	Unadjusted value of *p*	Adjusted eGFR^c^ (95% CI) WT homozygotes	Adjusted eGFR^c^ (95% CI) variant carriers	Adjusted^c^ value of *p*
*PPP3R1*^d^	rs875	2	A > G	32	0.017	49 (46–51)	54 (51–57)	0.009
*PPP3R1*^d^	rs12465425	2	T > G	32	0.013	49 (47–52)	54 (51–57)	0.010
*PPP3CC*	rs10108011	8	A > G	42	0.016	54 (50–57)	50 (48–53)	0.142
*PRKAG2*	rs7805747	7	G > A	24	0.012	52 (50–55)	50 (47–53)	0.219
*PLCB1*	rs170549	20	G > A	28	0.025	51 (49–55)	51 (48–54)	0.808

eGFR, estimated glomerular filtration rate; MAF, minor allele frequency; PLCB1, phospholipase C beta 1; PPP3CC, protein phosphatase 3 catalytic subunit gamma; PPP3R1, protein phosphatase 3 regulatory subunit B alpha; PRKAG2, protein kinase AMP-activated non-catalytic subunit gamma 2; SNV, single nucleotide variant; and WT, wild-type.

a Calculated using the MDRD formula.

b Major > minor alleles, respectively.

c Models adjusted for sex, pre-transplant eGFR, age at transplant, cyclosporine use at 1-year post-transplant, body mass index (BMI) at 1-year post-transplant, and the top four PCs.

d PPP3R1 SNVs are correlated (*r*^2^ = 0.91).

### Change in eGFR

**Figure 2:**
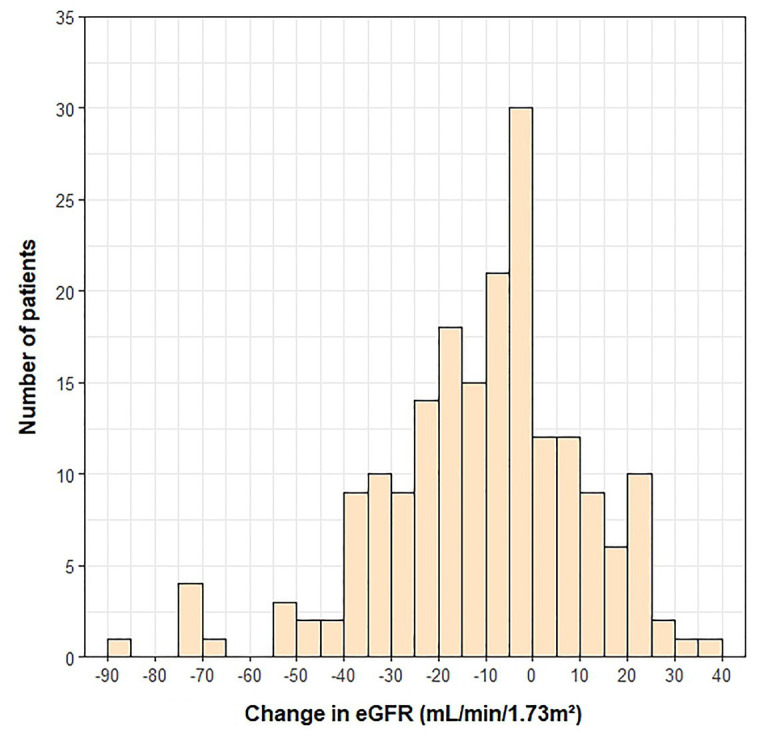
Histogram depicting the change in eGFR from pre-transplant to 1-year post-transplant.

The mean change in eGFR from pre-transplant to 1-year post-transplant was −10 ± 21 ml/min/1.73 m^2^ (Figure 2). Demographic and clinical variables associated with a greater decrease in eGFR and included in the multivariable models were presence of a LVAD prior to transplant (*p* < 0.001), absence of pre-transplant diabetes (*p* = 0.033), younger age at transplant (*p* < 0.001), and not being prescribed an ACE inhibitor or ARB 1-year post-transplant (*p* = 0.013). No SNVs were significantly associated with change in eGFR using an FDR of 20%. Four SNVs were suggestively associated with the outcome in univariate analyses (*p* < 0.05; Table 5) and were included in exploratory single-SNV multivariable models, adjusting for the covariates listed above and the top four PCs. Two SNVs (rs1800255 G > A and rs1878201 A > G) in the collagen type III alpha 1 chain (*COL3A1*) gene remained suggestively associated with change in eGFR from pre-transplant to 1-year post-transplant in the single-SNV adjusted models. Variant carriers had greater decline in eGFR in the 1st-year post-transplant vs. WT homozygotes for both SNVs. *COL3A1* rs1800255 and rs1878201 were no longer suggestively associated with the outcome in single-SNV adjusted analyses in non-Hispanic Americans of European ancestry (Supplementary Table S5).

**Table 5:** Unadjusted and adjusted single-SNV analyses for SNVs suggestively associated with change in eGFR^a^ from pre-transplant to 1-year post-transplant.

Gene name	rs number	Chromosome	Alleles^b^	MAF (%)	Unadjusted value of *p*	Adjusted change in eGFR^c^ (95% CI) WT homozygotes	Adjusted change in eGFR^c^ (95% CI) variant carriers	Adjusted^c^ value of *p*
*COL3A1**^d^*	rs1800255	2	G > A	23	0.008	−10 (−15 to −4)	−16 (−21 to −10)	0.041
*COL3A1**^d^*	rs1878201	2	A > G	24	0.010	−9 (−15 to −4)	−15 (−21 to −10)	0.042
*CACNA1D*	rs893365	3	C > T	49	0.022	−17 (−24 to −9)	−11 (−16 to −6)	0.117
*NR1I2*	rs2056530	3	C > T	26	0.029	−11 (−17 to −6)	−13 (−19 to −8)	0.542

CACNA1D, calcium voltage-gated channel subunit alpha1 D; COL3A1, collagen type III alpha 1 chain; eGFR, estimated glomerular filtration rate; MAF, minor allele frequency; NR1I2, nuclear receptor subfamily 1 group I member 2; SNV, single nucleotide variant; and WT, wild-type.

a Calculated using the MDRD formula.

b Major > minor alleles, respectively.

c Models adjusted for presence of a LVAD pre-transplant, pre-transplant diabetes, age at transplant, ACE inhibitor or ARB prescribed 1-year post-transplant, and the top four PCs.

d COL3A1 SNVs are correlated (*r*^2^ = 0.85).

## Discussion

In this study, we utilized a candidate gene approach to examine the association between 93 SNVs in CNI pharmacokinetic and pharmacodynamic genes and renal impairment following heart transplantation. Our study, in which the majority of patients were prescribed tacrolimus at the 1-year time point, is one of the largest and most comprehensive to date in this population. Two SNVs, *TGFB1* rs4803455 and *PLCB1* rs170549, were associated with renal dysfunction 1-year post-transplant after accounting for an FDR of 20%. TGFB-1, encoded by *TGFB1*, is a cytokine involved in the development of fibrosis, a key histological feature of chronic CNI exposure (Randhawa et al., 1993; Border and Noble, 1994). The expression of this important protein is modulated by the cell signaling enzyme, PLCB1 (Slattery et al., 2005).

We found variant A allele carriers of rs4803455 C > A, located in intron 2 of *TGFB1*, had lower odds of renal dysfunction compared to C/C homozygotes. TGFB-1 has been associated with increased fibrosis in multiple tissues, including the kidneys (Border and Noble, 1994). Importantly, two of the hallmarks of chronic renal dysfunction after transplantation are interstitial fibrosis and tubular atrophy (Myers et al., 1984; Randhawa et al., 1993). Intrarenal TGFB-1 expression has been shown to increase following administration of tacrolimus and cyclosporine (Khanna et al., 2002; Roos-van Groningen et al., 2006). Lachance et al. previously reported an association between *TGFB1* rs4803455 and eGFR in heart transplant recipients, of whom 21% were receiving tacrolimus and the average time post-transplant was 4.3 years (Lachance et al., 2012). However, that study did not describe which allele conveyed worse renal function, nor did it provide an estimate of the strength of the association. In non-transplant populations, variant A allele carriers of rs4803455 have been reported to have reduced risk of myopia, asthma, the formation of carotid plaque, breast cancer, and endometrial cancer (Zha et al., 2009; Wu et al., 2010; Deng et al., 2011; Boone et al., 2013; Yang et al., 2016). To date, rs4803455 has not been directly shown to influence *TGFB1* gene expression or protein function. Due to its location in intron 2, further investigation is warranted to identify if a functional variant in linkage disequilibrium with rs4803455 is driving the observed associations.

Other studies have reported an association between the *TGFB1* missense variant, Leu10Pro (L10P), and renal function in heart transplant recipients (Baan et al., 2000; Lacha et al., 2001; van de Wetering et al., 2006). Codon 10 proline carriers had a higher risk of renal dysfunction in patients taking CNIs compared to leucine/leucine patients in two studies, whereas Lacha et al. reported worse progression of renal dysfunction in leucine carriers (Baan et al., 2000; Lacha et al., 2001; van de Wetering et al., 2006). We found no association between L10P and renal function outcomes at 1-year post-transplant in our cohort. Our results agree with Klauke et al. (2008) and Lachance et al. (2012) both of whom found no association between L10P and renal function in heart transplant recipients. Possible reasons for the discrepant results between studies include the variable post-transplant time points analyzed and differing definitions of renal dysfunction. Nonetheless, our results, in combination with previous findings, suggest that SNVs in *TGFB1* may play a role in the development of renal dysfunction following heart transplantation.

We also found that carriers of the intronic *PLCB1* rs170549 variant A allele had higher odds of renal dysfunction when compared to individuals with the G/G genotype. PLCB1 catalyzes the formation of diacylglycerol and is expressed in multiple tissues, including vascular smooth muscle cells (Schelling et al., 1997; Hisatsune et al., 2005). In turn, diacylglycerol activates protein kinase C, which regulates a variety of cellular targets *via* phosphorylation (Nishizuka, 1988). Protein kinase C has been shown to modulate cyclosporine-induced up-regulation of TGFB-1 and inhibition of endothelial nitric oxide synthase by tacrolimus (Slattery et al., 2005; Chiasson et al., 2011). Furthermore, *PLCB1* rs170549 has previously been associated with eGFR in adult heart transplant recipients, although the study did not describe which allele was associated with worse renal function, nor the magnitude of the association (Lachance et al., 2012). Given that rs170549 is located in an intron, it may be in linkage disequilibrium with another functional variant in *PLCB1*, which could influence its protein structure and/or expression. Thus, additional studies are needed to elucidate the mechanism by which *PLCB1* rs170549, or a functional SNV linked with this intronic variant, influences the development of renal dysfunction in heart transplant recipients receiving CNIs.

In exploratory analyses, we found carriers of the *CACNA1D* rs893365 variant T allele had lower odds of renal dysfunction 1-year post-transplant. *CACNA1D* encodes a subunit of the calcium channel, Ca_v_1.3, which plays an important role in the production and secretion of aldosterone from the adrenal cortex (Yang et al., 2020). Aldosterone increases sodium and fluid retention which, in turn, results in higher blood pressure as part of renin-angiotensin-aldosterone system (RAS) activation. RAS activation leads to vasoconstriction of renal afferent and efferent arterioles, changes which are also observed following CNI treatment (Kaye et al., 1993; Textor et al., 1995; Mennuni et al., 2014). Another intronic variant in *CACNA1D*, rs9810888, has previously been associated with the presence of hypertension in non-transplanted adults (Lu et al., 2015). As such, genetic variation in *CACNA1D* may increase the odds of developing hypertension, a condition that contributes to the occurrence of renal dysfunction after heart transplantation and which is exacerbated by CNI use (Lindenfeld et al., 2004). We also found *PRKAG2* rs7805747 variant A carriers had higher odds of renal impairment 1-year after transplant than G/G homozygotes. *PRKAG2* encodes the gamma-2 subunit of AMP-activated protein kinase, an enzyme that regulates metabolism during cellular stress (Hardie et al., 1998). Furthermore, AMP-activated protein kinase may protect against the development of tubulointerstitial renal fibrosis, a common histological finding in patients treated with CNIs, through the inhibition TGFB-1 (Naesens et al., 2009; Lee et al., 2013). *In silico* data suggest *PRKAG2* rs7805747 could affect the binding of transcription factors, such as hepatocyte nuclear factor 4 alpha, to *PRKAG2* (Wang et al., 2018). Additionally, the *PRKAG2* rs7805747 variant A allele has been associated with higher odds of renal dysfunction in genome-wide association studies in non-transplant populations (Kottgen et al., 2010; Pattaro et al., 2016). Given the exploratory nature of our analyses, the lack of *in vitro* data describing the functional significance of *CACNA1D* rs893365 and *PRKAG2* rs7805747, and the intronic location of both variants, further studies are necessary to determine how these SNVs, or linked functional variants, impact renal function in heart transplant recipients prescribed CNIs.

There are limitations to our study that deserve to be acknowledged. First, there was a broad range of dates of transplant in our cohort (1989–2016). Patient care during this period has changed, and some of these changes might have influenced renal dysfunction (e.g., type of CNI used, CNI goal ranges). Second, it is likely that survival bias is present, with some patients with renal dysfunction dying prior to study enrollment. Furthermore, we did not include patients with severe renal impairment whose CNI was discontinued in favor of an mTOR inhibitor (i.e., sirolimus or everolimus), which may also bias our results. Third, multiple suggestive SNVs from the whole cohort were not associated with the primary and secondary outcomes when the analysis was limited to non-Hispanic Americans of European ancestry. While this may be due, in part, to a reduction in power, we cannot exclude the possibility that population stratification may have impacted these findings. Fourth, the functional effects of many of the significant and suggestive SNVs have not been reported in the literature. Future studies should focus on replicating these findings and determining the mechanistic underpinnings of their effects. Fifth, the two SNVs significantly associated with renal dysfunction 1-year post-transplant in our cohort were also associated with pre-transplant renal dysfunction. While these SNVs continued to be associated with the primary outcome after accounting for pre-transplant renal impairment in multi-SNV adjusted models, it is possible that the effects of these variants on renal function post-transplant may exist independent of CNI exposure. Finally, we used a single eGFR measurement in our primary and secondary outcomes. Although this is a common approach to assess renal function post-transplant, it may not capture other acute processes that may have occurred around that time point (e.g., acute kidney injury, dehydration; Sanchez-Lazaro et al., 2015; Asleh et al., 2018).

In conclusion, we provide additional evidence that SNVs in the pro-fibrotic *TGFB1* and cell signaling *PLCB1* genes may play a role in the development of renal dysfunction in adult heart transplant recipients taking CNIs. Novel genetic markers, such as *TGFB1* rs4803455 and *PLCB1* rs170549, may serve as tools that clinicians can utilize to assess a patient’s risk of CNI-associated renal dysfunction following heart transplantation, thereby improving clinical care and advancing precision medicine in this population.

## Data Availability Statement

The datasets for this article are not publicly available due to concerns regarding participant/patient anonymity. Requests to access the datasets should be directed to the corresponding author.

## Ethics Statement

The studies involving human participants were reviewed and approved by Colorado Multiple Institutional Review Board. The patients/participants provided their written informed consent to participate in this study.

## Author Contributions

CA conceptualized the study idea. KO, CA, and KD designed the study and collected and analyzed the data. LS and NR aided in data analysis. AA, JL, and RP were involved in patient recruitment and provided clinical input. KO and CA wrote the manuscript. All authors contributed to the article and approved the submitted version.

### Conflict of Interest

The authors declare that the research was conducted in the absence of any commercial or financial relationships that could be construed as a potential conflict of interest.

## Footnotes

^1^www.pharmgkb.org
