# Supplementary material for: Association Between Variants in Calcineurin Inhibitor Pharmacokinetic and Pharmacodynamic Genes and Renal Dysfunction in Adult Heart Transplant Recipients
Source: Front Genet. 2021 Apr 1;12:658983. doi: 10.3389/fgene.2021.658983 (PMC8047196; doi:10.3389/fgene.2021.658983)
Supplement: Supplementary file 1 [file Data_Sheet_1.docx]

**Figure S1.** Scree plot of 292,277 single nucleotide variants used to select the number of principal components to include for the entire cohort (N=192)

**
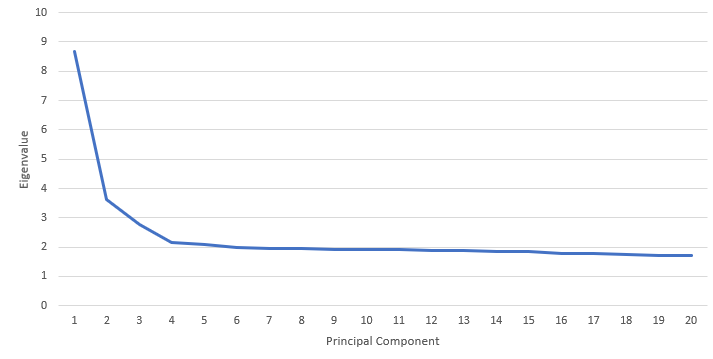
**

**TABLE S1.** Single nucleotide variants investigated (n=93)

| **Gene** | **rs number** | **Alleles^a^** | **MAF^b^** | **Region** | **On CU-MEGA^EX^ or Imputed** | **Imputation r^2^** | **Chromosome** | **Position^c^** |
| --- | --- | --- | --- | --- | --- | --- | --- | --- |
| Cytochrome P450 family 2 subfamily J member 2 (*CYP2J2*) | rs890293 | C>A | 11% | Promoter | Imputed | 0.962 | 1 | 60392494 |
| Renin (*REN*) | rs5707 | A>C | 25% | Intron | CU-MEGA^EX^ | - | 1 | 204129671 |
| Interleukin 10  (*IL-10*) | rs1800872 | G>T | 26% | Promoter | Imputed | 0.945 | 1 | 206946407 |
| Angiotensinogen (*AGT*) | rs7079 | G>T | 26% | 3’ UTR | CU-MEGA^EX^ | - | 1 | 230838331 |
| Angiotensinogen (*AGT*) | rs3789664 | C>T | 5% | Intron | Imputed | 0.852 | 1 | 230843424 |
| Angiotensinogen (*AGT*) | rs2478544 | C>G | 19% | Intron | Imputed | 0.908 | 1 | 230844196 |
| Angiotensinogen (*AGT*) | rs699 | G>A | 48% | Non-synonymous (Thr268Met) | CU-MEGA^EX^ | - | 1 | 230845794 |
| Angiotensinogen (*AGT*) | rs4762 | G>A | 10% | Non-synonymous (Thr207Met) | Imputed | 0.835 | 1 | 230845977 |
| Angiotensinogen (*AGT*) | rs5051 | T>C | 48% | Promoter | CU-MEGA^EX^ | - | 1 | 230849872 |
| Protein phosphatase 3 regulatory subunit B, alpha (*PPP3R1)* | rs875 | A>G | 32% | 3’ UTR | Imputed | 0.924 | 2 | 68407926 |
| Protein phosphatase 3 regulatory subunit B, alpha (*PPP3R1)* | rs1868402 | A>G | 23% | Intron | CU-MEGA^EX^ | - | 2 | 68409037 |
| Protein phosphatase 3 regulatory subunit B, alpha (*PPP3R1)* | rs7599591 | A>C | 26% | Intron | Imputed | 0.814 | 2 | 68412906 |
| Protein phosphatase 3 regulatory subunit B, alpha (*PPP3R1)* | rs12465425 | T>G | 32% | Intron | Imputed | 0.881 | 2 | 68470425 |
| Protein phosphatase 3 regulatory subunit B, alpha (*PPP3R1)* | rs13035506 | G>C | 25% | Intron | Imputed | 0.850 | 2 | 68477609 |
| Collagen type III alpha I chain (*COL3A1*) | rs1878201 | A>G | 24% | Intron | Imputed | 0.914 | 2 | 189841613 |
| Collagen type III alpha I chain (*COL3A1*) | rs1800255 | G>A | 23% | Non-synonymous (Ala698Thr) | Imputed | 0.919 | 2 | 189864080 |
| UDP Glucuronosyltransf-erase family 1 member A1 (*UGT1A1*) | rs3771342 | G>T | 11% | Intron | CU-MEGA^EX^ | - | 2 | 234672663 |
| XPC complex subunit, DNA damage recognition and repair factor (*XPC*) | rs2228001 | T>G | 43% | Non-synonymous (Lys939Gln) | Imputed | 0.982 | 3 | 14187449 |
| Toll like receptor 9 (*TLR9*) | rs352139 | C>T | 45% | Intron | CU-MEGA^EX^ | - | 3 | 52258372 |
| Calcium voltage-gated channel subunit alpha1 D (*CACNA1D*) | rs893365 | C>T | 49% | Intron | Imputed | 0.947 | 3 | 53841146 |
| Nuclear receptor subfamily 1 group I member 2 (*NR1I2*) | rs12488820 | C>T | 45% | Intron | Imputed | 0.943 | 3 | 119502069 |
| Nuclear receptor subfamily 1 group I member 2 (*NR1I2*) | rs2056530 | C>T | 26% | Intron | Imputed | 0.906 | 3 | 119506462 |
| Nuclear receptor subfamily 1 group I member 2 (*NR1I2*) | rs1403526 | A>G | 33% | Intron | Imputed | 0.889 | 3 | 119509381 |
| Nuclear receptor subfamily 1 group I member 2 (*NR1I2*) | rs2472677 | T>C | 42% | Intron | Imputed | 0.944 | 3 | 119518417 |
| Nuclear receptor subfamily 1 group I member 2 (*NR1I2*) | rs13059232 | C>T | 38% | Intron | Imputed | 0.948 | 3 | 119521055 |
| Nuclear receptor subfamily 1 group I member 2 (*NR1I2*) | rs2461818 | C>T | 9% | Intron | Imputed | 0.862 | 3 | 119523872 |
| Nuclear receptor subfamily 1 group I member 2 (*NR1I2*) | rs2472681 | T>C | 34% | Intron | Imputed | 0.927 | 3 | 119529689 |
| Nuclear receptor subfamily 1 group I member 2 (*NR1I2*) | rs2472682 | C>A | 44% | Intron | Imputed | 0.901 | 3 | 119532652 |
| Nuclear receptor subfamily 1 group I member 2 (*NR1I2*) | rs2276707 | C>T | 22% | Intron | Imputed | 0.977 | 3 | 119534153 |
| Nuclear receptor subfamily 1 group I member 2 (*NR1I2*) | rs10511395 | C>A | 16% | 3’ UTR | Imputed | 0.980 | 3 | 119536559 |
| Nuclear receptor subfamily 1 group I member 2 (*NR1I2*) | rs3814057 | A>C | 23% | 3’ UTR | CU-MEGA^EX^ | - | 3 | 119537254 |
| Angiotensin II receptor type 1 (*AGTR1*) | rs388915 | A>G | 31% | Intron | Imputed | 0.919 | 3 | 148447756 |
| Angiotensin II receptor type 1 (*AGTR1*) | rs5186 | A>C | 25% | 3’ UTR | CU-MEGA^EX^ | - | 3 | 148459988 |
| Membrane metalloendopeptidase (*MME)* | rs16824656 | A>G | 8% | Intron | Imputed | 0.860 | 3 | 154898127 |
| Corin, serine peptidase (*CORIN*) | rs11934749 | C>T | 13% | Non-synonymous (Arg525His) | CU-MEGA^EX^ | - | 4 | 47667064 |
| Shroom family member 3 (*SHROOM3*) | rs17319721 | G>A | 38% | Intron | CU-MEGA^EX^ | - | 4 | 77368847 |
| Alcohol dehydrogenase 4 (class II), pi polypeptide (*ADH4*) | rs1126672 | G>A | 25% | Synonymous (Leu370=) | CU-MEGA^EX^ | - | 4 | 100047812 |
| Protein phosphatase 3 catalytic subunit alpha (*PPP3CA)* | rs17030727 | A>C | 11% | Intron | Imputed | 0.627 | 4 | 101963249 |
| Protein phosphatase 3 catalytic subunit alpha (*PPP3CA)* | rs1395475 | C>T | 15% | Intron | CU-MEGA^EX^ | - | 4 | 102115186 |
| Protein phosphatase 3 catalytic subunit alpha (*PPP3CA)* | rs3811063 | G>A | 44% | Intron | Imputed | 0.903 | 4 | 102120290 |
| Protein phosphatase 3 catalytic subunit alpha (*PPP3CA)* | rs2659522 | C>T | 10% | Intron | Imputed | 0.885 | 4 | 102147182 |
| Protein phosphatase 3 catalytic subunit alpha (*PPP3CA)* | rs13137393 | T>C | 33% | Intron | Imputed | 0.868 | 4 | 102171725 |
| Protein phosphatase 3 catalytic subunit alpha (*PPP3CA)* | rs2850969 | T>C | 17% | Intron | CU-MEGA^EX^ | - | 4 | 102183594 |
| Protein phosphatase 3 catalytic subunit alpha (*PPP3CA)* | rs2850343 | C>T | 11% | Intron | Imputed | 0.671 | 4 | 102197855 |
| Nuclear receptor subfamily 3 group C member 2 (*NR3C2*) | rs1490453 | G>A | 18% | Intron | CU-MEGA^EX^ | - | 4 | 149321346 |
| Nuclear receptor subfamily 3 group C member 2 (*NR3C2*) | rs4635799 | T>C | 46% | Intron | CU-MEGA^EX^ | - | 4 | 149350527 |
| 5-Methyltetrahydrofo-late-homocysteine methyltransferase reductase (*MTRR*) | rs1532268 | C>T | 31% | Non-synonymous (Ser175Leu) | CU-MEGA^EX^ | - | 5 | 7878179 |
| 5-Methyltetrahydrofo-late-homocysteine methyltransferase reductase (*MTRR*) | rs1802059 | G>A | 24% | Synonymous (Ala637=) | Imputed | 0.696 | 5 | 7897319 |
| Adrenoceptor beta 2 (*ADRB2*) | rs1042713 | G>A | 39% | Non-synonymous (Gly16Arg) | CU-MEGA^EX^ | - | 5 | 148206440 |
| Adrenoceptor beta 2 (*ADRB2*) | rs1042714 | C>G | 38% | Non-synonymous (Gln27Glu) | Imputed | 0.804 | 5 | 148206473 |
| Endothelin 1 (*EDN1*) | rs5370 | G>T | 19% | Non-synonymous (Lys198Asn) | CU-MEGA^EX^ | - | 6 | 12296255 |
| ATP binding cassette subfamily B member 1 (*ABCB1*) | rs1045642 | G>A | 45% | Synonymous (Ile1145=) | CU-MEGA^EX^ | - | 7 | 87138645 |
| ATP binding cassette subfamily B member 1 (*ABCB1*) | rs1128503 | G>A | 41% | Synonymous (Gly412=) | CU-MEGA^EX^ | - | 7 | 87179601 |
| Caveolin 1 (*CAV1*) | rs4730751 | C>A | 25% | Intron | Imputed | 0.918 | 7 | 116180850 |
| Paired box 4 (*PAX4*) | rs712704 | A>G | 21% | Promoter | Imputed | 0.899 | 7 | 127258208 |
| Nitric oxide synthase 3 (*NOS3*) | rs2070744 | T>C | 40% | Intron | Imputed | 0.798 | 7 | 150690079 |
| Nitric oxide synthase 3 (*NOS3*) | rs1799983 | G>T | 32% | Non-synonymous (Glu298Asp) | Imputed | 0.817 | 7 | 150696111 |
| Protein kinase AMP-activated non-catalytic subunit gamma 2 (*PRKAG2*) | rs7805747 | G>A | 24% | Intron | Imputed | 0.968 | 7 | 151407801 |
| CUB and Sushi multiple domains 1 (*CSMD1*) | rs13270945 | A>G | 38% | Intron | CU-MEGA^EX^ | - | 8 | 4045470 |
| CUB and Sushi multiple domains 1 (*CSMD1*) | rs9644363 | G>C | 36% | Intron | Imputed | 0.869 | 8 | 4045591 |
| CUB and Sushi multiple domains 1 (*CSMD1*) | rs34851282 | A>C | 35% | Intron | Imputed | 0.837 | 8 | 4045952 |
| Protein phosphatase 3 catalytic subunit gamma (*PPP3CC)* | rs7821470 | T>C | 13% | Intron | CU-MEGA^EX^ | - | 8 | 22305501 |
| Protein phosphatase 3 catalytic subunit gamma (*PPP3CC)* | rs10108011 | A>G | 42% | Intron | Imputed | 0.933 | 8 | 22320806 |
| Protein phosphatase 3 catalytic subunit gamma (*PPP3CC)* | rs2461494 | A>G | 20% | Intron | Imputed | 0.672 | 8 | 22349511 |
| Protein phosphatase 3 catalytic subunit gamma (*PPP3CC)* | rs2449348 | C>T | 47% | Intron | Imputed | 0.901 | 8 | 22359572 |
| Natriuretic peptide receptor 2 (*NPR2)* | rs10758325 | G>A | 46% | Intron | Imputed | 0.825 | 9 | 35804149 |
| G protein subunit alpha q (*GNAQ*) | rs1977472 | G>C | 35% | Intron | Imputed | 0.794 | 9 | 80351444 |
| G protein subunit alpha q (*GNAQ*) | rs1059033 | A>G | 42% | Intron | Imputed | 0.737 | 9 | 80506471 |
| G protein subunit alpha q (*GNAQ*) | rs4745683 | C>T | 23% | Intron | Imputed | 0.955 | 9 | 80592519 |
| G protein subunit alpha q (*GNAQ*) | rs1930544 | T>C | 30% | Intron | Imputed | 0.827 | 9 | 80624691 |
| Protein phosphatase 3 catalytic subunit beta (*PPP3CB)* | rs12644 | C>T | 14% | 3’ UTR | CU-MEGA^EX^ | - | 10 | 75197051 |
| Protein phosphatase 3 catalytic subunit beta (*PPP3CB)* | rs12775630 | A>T | 5% | Intron | CU-MEGA^EX^ | - | 10 | 75203331 |
| Cytochrome P450 family 2 subfamily C member 8 (*CYP2C8*) | rs10509681 | T>C | 14% | Non-synonymous (Lys399Arg) | CU-MEGA^EX^ | - | 10 | 96798749 |
| ATP binding cassette subfamily C member 2 (*ABCC2*) | rs717620 | C>T | 22% | 5’ UTR | CU-MEGA^EX^ | - | 10 | 101542578 |
| ATP binding cassette subfamily C member 2 (*ABCC2*) | rs3740066 | C>T | 40% | Synonymous (Ile1324=) | CU-MEGA^EX^ | - | 10 | 101604207 |
| Adrenoreceptor beta 1 (*ADRB1*) | rs1801252 | A>G | 17% | Non-synonymous (Ser49Gly) | Imputed | 0.947 | 10 | 115804036 |
| Adrenoreceptor beta 1 (*ADRB1*) | rs1801253 | C>G | 29% | Non-synonymous (Arg389Gly) | Imputed | 0.770 | 10 | 115805056 |
| Solute carrier organic anion transporter family member 1B1 (*SLCO1B1*) | rs2306283 | A>G | 46% | Non-synonymous (Asn130Asp) | CU-MEGA^EX^ | - | 12 | 21329738 |
| Solute carrier organic anion transporter family member 1B1 (*SLCO1B1*) | rs11045819 | C>A | 15% | Non-synonymous (Pro155Thr) | CU-MEGA^EX^ | - | 12 | 21329813 |
| Solute carrier organic anion transporter family member 1B1 (*SLCO1B1*) | rs4149056 | T>C | 15% | Non-synonymous (Val174Ala) | CU-MEGA^EX^ | - | 12 | 21331549 |
| Intergenic region | rs3811321 | A>C | 8% | Intergenic | CU-MEGA^EX^ | - | 14 | 22481824 |
| Methenyltetrahydr-ofolate synthetase (*MTHFS*) | rs6495446 | C>T | 34% | Intron | Imputed | 0.966 | 15 | 80154982 |
| ATP binding cassette subfamily C member 1 (*ABCC1*) | rs2074086 | T>C | 38% | Intron | Imputed | 0.953 | 16 | 16181142 |
| Uromodulin (*UMOD*) | rs12917707 | G>T | 14% | Promoter | Imputed | 0.987 | 16 | 20367690 |
| Protein kinase C beta (*PRKCB*) | rs3760106 | C>T | 19% | Promoter | CU-MEGA^EX^ | - | 16 | 23845796 |
| Protein kinase C beta (*PRKCB*) | rs2575390 | C>G | 22% | Promoter | Imputed | 0.823 | 16 | 23846754 |
| Protein kinase C beta (*PRKCB*) | rs11074606 | A>G | 36% | Intron | Imputed | 0.959 | 16 | 24132569 |
| Gigaxonin (*GAN*) | rs2608555 | C>T | 20% | Synonymous (Tyr431=) | Imputed | 0.808 | 16 | 81398635 |
| Nuclear factor of activated T cells 1 (*NFATC1*) | rs2280055 | C>T | 18% | Intron | Imputed | 0.990 | 18 | 77217924 |
| Transforming growth factor beta 1 (*TGFB1*) | rs4803455 | C>A | 45% | Intron | CU-MEGA^EX^ | - | 19 | 41851509 |
| Transforming growth factor beta 1 (*TGFB1*) | rs1800470 | A>G | 41% | Non-synonymous (Leu10Pro) | Imputed | 0.899 | 19 | 41858921 |
| Phospholipase C beta 1 (*PLCB1*) | rs227129 | G>A | 28% | Intron | Imputed | 0.841 | 20 | 8433588 |
| Phospholipase C beta 1 (*PLCB1*) | rs170549 | G>A | 28% | Intron | Imputed | 0.758 | 20 | 8438226 |

Abbreviations: CU-MEGA^EX^, Customized Expanded Multi-Ethnic Global Array; MAF, minor allele frequency; UTR, untranslated region. ^a^Major>minor alleles, respectively. ^b^Minor allele frequency in the entire cohort (N=192). ^c^Per Genome Reference Consortium Human Build 37.

**Table S2** Multiple-SNV adjusted model for SNVs significantly associated with renal dysfunction^a^ one-year post-transplant in non-Hispanic Americans of European ancestry (N=147)

| Gene name | rs number | Chromosome | Alleles^b^ | Reference group | Adjusted^c^ odds ratio (95% CI) | Adjusted^c^ p-value |
| --- | --- | --- | --- | --- | --- | --- |
| *TGFB1* | rs4803455 | 19 | C>A | C/C | 0.39 (0.17-0.90) | 0.027 |
| *PLCB1* | rs170549 | 20 | G>A | G/G | 2.52 (1.10-5.77) | 0.029 |

Abbreviations: *PLCB1*, phospholipase C beta 1; SNV, single nucleotide variant; *TGFB1*, transforming growth factor beta 1. ^a^Defined as an eGFR <45 mL/min/1.73m^2^. ^b^Major>minor alleles, respectively. ^c^Model adjusting for pre-transplant renal dysfunction, age at transplant, cyclosporine use at one-year post-transplant, and transplant era.

**Table S3** Adjusted single-SNV analyses for SNVs suggestively associated with renal dysfunction^a^ at one-year post-transplant in non-Hispanic Americans of European ancestry (N=147)

| Gene name | rs number | Chromosome | Alleles^b^ | Reference group | Adjusted^c^ odds ratio (95% CI) | Adjusted^c^ p-value |
| --- | --- | --- | --- | --- | --- | --- |
| *CACNA1D* | rs893365 | 3 | C>T | CC | 0.21 (0.08-0.57) | 0.002 |
| *PPP3CC* | rs10108011 | 8 | A>G | AA | 2.20 (0.92-5.23) | 0.075 |
| *PPP3CC* | rs2461494 | 8 | A>G | AA | 2.17 (0.98-4.83) | 0.057 |
| *PRKAG2* | rs7805747 | 7 | G>A | GG | 2.52 (1.16-5.49) | 0.020 |
| *NR3C2* | rs1490453 | 4 | G>A | GG | 0.42 (0.18-1.01) | 0.052 |

Abbreviations: *CACNA1D*, calcium voltage-gated channel subunit alpha1 D; *NR3C2*, nuclear receptor subfamily 3 group C member 2; *PPP3CC*, protein phosphatase 3 catalytic subunit gamma; *PRKAG2*, protein kinase AMP-activated non-catalytic subunit gamma 2; SNV, single nucleotide variant. ^a^Defined as an eGFR <45 mL/min/1.73m^2^. ^b^Major>minor alleles, respectively. ^c^Models adjusted for pre-transplant renal dysfunction, age at transplant, cyclosporine use at one-year post-transplant, and transplant era.

**Table S4** Adjusted single-SNV analyses for SNVs suggestively associated with eGFR^a^ at one-year post-transplant in non-Hispanic Americans of European ancestry (N=147)

| Gene name | rs number | Chromosome | Alleles^b^ | Adjusted eGFR^c^ (95% CI)  WT homozygotes | Adjusted eGFR^c^ (95% CI)  Variant carriers | Adjusted^c^  p-value |
| --- | --- | --- | --- | --- | --- | --- |
| *PPP3R1* | rs875 | 2 | A>G | 48 (45-51) | 51 (48-55) | 0.087 |
| *PPP3R1* | rs12465425 | 2 | T>G | 47 (45-51) | 51 (48-55) | 0.076 |

Abbreviations: eGFR, estimated glomerular filtration rate; *PPP3R1*, protein phosphatase 3 regulatory subunit B alpha; SNV, single nucleotide variant; WT, wild-type. ^a^Calculated using the Modification of Diet in Renal Disease (MDRD) formula. ^b^Major>minor alleles, respectively. ^c^Models adjusted for sex, pre-transplant eGFR, age at transplant, cyclosporine use at one-year post-transplant, and body mass index at one-year post-transplant.

**Table S5** Adjusted single-SNV analyses for SNVs suggestively associated with change in eGFR^a^ from pre-transplant to one-year post-transplant in non-Hispanic Americans of European ancestry (N=147)

| Gene name | rs number | Chromosome | Alleles^b^ | Adjusted change in eGFR^c^ (95% CI) – WT homozygotes | Adjusted change in eGFR^c^ (95% CI) – Variant carriers | Adjusted^c^ p-value |
| --- | --- | --- | --- | --- | --- | --- |
| *COL3A1* | rs1800255 | 2 | G>A | -12 (-18 to -5) | -16 (-22 to -9) | 0.163 |
| *COL3A1* | rs1878201 | 2 | A>G | -12 (-18 to -6) | -15 (-21 to -9) | 0.345 |

Abbreviations: *COL3A1*, collagen type III alpha 1 chain; eGFR, estimated glomerular filtration rate; SNV, single nucleotide variant; WT, wild-type. ^a^Calculated using the Modification of Diet in Renal Disease (MDRD) formula. ^b^Major>minor alleles, respectively. ^c^Models adjusted for presence of a left ventricular assist device pre-transplant, pre-transplant diabetes, age at transplant, and ACE inhibitor or ARB prescribed one-year post-transplant.
